# Quantification of the Blood Platelet Reactivity in the ADP-Induced Model of Non-Lethal Pulmonary Thromboembolism in Mice with the Use of Laser Doppler Flowmetry

**DOI:** 10.1371/journal.pone.0146346

**Published:** 2016-01-11

**Authors:** Tomasz Przygodzki, Marcin Talar, Agnieszka Blazejczyk, Vyacheslav Kalchenko, Cezary Watala

**Affiliations:** 1 Department of Haemostasis and Haemostatic Disorders, Chair of Biomedical Sciences, Medical University of Lodz, Lodz, Poland; 2 Ludwik Hirszfeld Institute of Immunology and Experimental Therapy, Polish Academy of Sciences, Wroclaw, Poland; 3 Department of Veterinary Resources, Weizmann Institute of Science, Rehovot, Israel; Singapore Immunology Network, SINGAPORE

## Abstract

**Introduction:**

The paper describes an alternative method for quantification of *in vivo* ADP-induced thromboembolism. The aim of the studies was to develop a method of quantification which would not require either extravasation or labelling of platelets. Our proposed approach is based on the monitoring of changes of blood flow with the use of laser Doppler flowmetry.

**Materials and Methods:**

Mice of C57Bl strain were used in the study. ADP was injected to the vena cava and blood flow was monitored with the use of a laser Doppler flowmeter in the mesentery. Measurements in platelet-depleted mice, mice pretreated with cangrelor, an ADP receptor antagonist, and eptifibatide, a blocker of fibrinogen binding to GPIIbIIIa, were conducted as the proof-of-concept in the performed experiments. Intravital microscopy and *ex vivo* imaging of organs was performed to identify the sites of aggregate formation resulting from ADP injection.

**Results:**

The injection of ADP resulted in a dose-dependent reduction of the blood flow in the mesentery. These responses were fully attributable to blood platelet aggregation, as shown by the lack of the effect in platelet-depleted mice, and significantly reduced responses in mice pretreated with cangrelor and eptifibatide. No platelet aggregate formation in mesenteric vessels was revealed by intravital microscopy, while *ex vivo* imaging showed accumulation of fluorescent labelled platelets in the lung.

**Conclusions:**

Injection of ADP to the venous system results in the formation of platelet aggregates predominantly in the lung. This results in reversible blood flow cessation in peripheral blood vessels. The measurement of this blood flow cessation in the mesentery allows indirect measurement of ADP-induced pulmonary thromboembolism. We suggest that this approach can be useful for *in vivo* screening for antiplatelet drug candidates.

## Introduction

Several methods for studying *in vivo* blood platelet reactivity in animal models have been developed. The most commonly used techniques are based on the experimental disruption of endothelial layer, which inevitably leads to the exposure of the subendothelial matrix, thus triggering blood platelet activation and adherence to subendothelial matrix components, and ultimately, thrombus formation [1,2].

However, experimental approaches aimed at studying platelet activation under conditions of an intact endothelial layer have been developed. These are based on either activation of endothelial layer with the use of a laser beam [3] or by systemic injection of a platelet agonist.

In the latter case, the venous injection of the agonist results in the formation of occlusive thrombi [4]. Two methods that allow this effect to be monitored *in vivo* are described in the literature. In one of them, the measure of platelet reactivity is the death rate of the treated animals, which is believed to result from the formation of thrombi in lungs [5]. Quantification of the response to agonist in this method is limited. The second method is based on the infusion of radiolabeled platelets to the animal circulation and monitoring the rate at which radiolabeled platelets accumulate in lung vessels in response to the non-lethal doses of agonist [4,6,7]. The main limitations of this technique include the need for access to an isotopic facility and the disposal of radioactive animal remains. The additional drawback of this method is that the platelets are extravasated prior to radiolabelling, which inevitably causes the risk of artefactual activation.

The present study tests an alternative method for monitoring *in vivo* agonist-induced platelet aggregation, which requires neither platelet extravasation nor labelling. Since platelet aggregates can occlude small vessels and thus impair blood flow in a vascular bed, we hypothesized that platelet aggregation induced *in vivo* can be monitored by the instant measuring of blood flow in this vascular bed. Among the various methods used to monitor blood flow *in vivo*, Laser Doppler Flowmetry (LDF) was chosen, as it allows various vascular beds or blood vessels to be operated on, depending on the researcher’s needs. The basic principles of LDF are as follows: a beam of laser light is directed by an optical fiber to the probe head. When the probe head is applied to a blood vessel, all blood cells traversing this volume are struck by the laser light, partly reflecting it, which then undergoes a Doppler shift. The reflected light is a mixture of non-shifted and Doppler-shifted components, the magnitude and frequency distribution of the latter being related to the number and velocity of moving blood cells within the volume of tissue.

An intravital injection of ADP was chosen to test the proposed approach on the basis of previous studies which show that this particular agonist causes reversible platelet aggregation *in vivo* [6,7]. Of the several vascular beds used in previous studies of *in vivo* thrombus formation, one based on mesenteric vessels was the most suitable. The mesentery can be easily immobilized by attaching to a surface and is also isolated from the respiratory movements of the animal that are a common and undesired source of noise in LDF measurements. To provide a proof-of-concept of the method, the mice with induced thrombocytopenia were compared with mice pretreated with cangrelor, the antagonist of P_2_Y_12_ receptor for ADP, and with eptifibatide, the inhibitor of fibrinogen binding to its receptor.

## Materials and Methods

### LDF measurements

The studies were performed with the use of a ML191 Blood Flowmeter (ADInstruments, US) equipped with a temperature stabilized semiconductor laser diode with emission at 830 ± 10 nm. Measurements were conducted by means of a standard pencil probe connected to the flowmeter through two optic fibers, each of 125 μm in diameter, separated by 300 μm. Maximum laser power at probe tip is 1 mW, according to manufacturer. Doppler shift was analyzed and recalculated to LDF output signal by the flowmeter and transferred to ML870 PowerLab 8/30 data acquisition system. LDF was recorded as a function of time by Chart 5 software.

**Fig 1:**
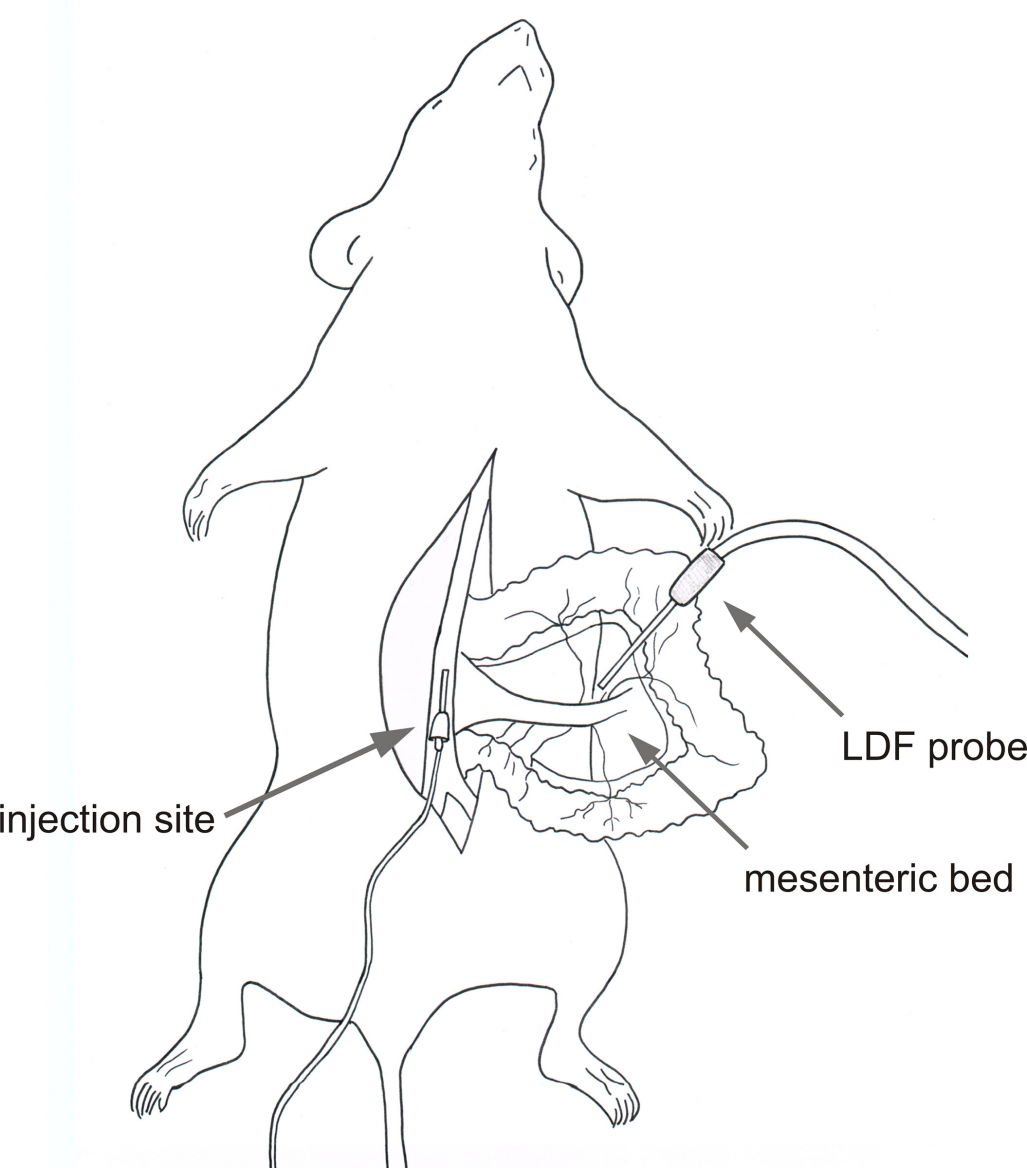
Schematic representation of the experimental setup used in the study. The anesthetized mouse in a supine position is located on a heated surgical table. The mesenteric bed is prepared. ADP solution is injected into the *vena cava*. Blood flow is measured in the mesenteric bed with the use of a laser Doppler flow probe.

Male C57BL/6J mice were used as subjects. The animals were anesthetized with an intramuscular injection of ketamine and sedazine and placed on a surgical table, ensuring control of body temperature. The abdominal cavity of the anesthetized mouse was opened, and the mesenteric bed was gently exposed with the use of cotton swabs and fixed on a Petri dish. A laser Doppler probe was fixed in a holder to allow positioning and mechanical stabilization, and placed over one of the branches including the second-order mesenteric vein and artery. The scheme of the experimental setup is presented on **Fig 1**. The distance between the tip of the probe and the bundle of vessels was approximately 2 mm. The abdominal vena cava was cannulated with the use of a 22G plastic catheter. ADP, dissolved at a concentration of 2.5 mg/ml in saline, was infused into the abdominal *vena cava* using an infusion pump (BBraun, Germany) for 10 sec with the maintenance of the flow rates resulting in final doses of 0.5, 1, 2, 3.5, 5 and 10 mg/kg b.w. Consecutive injections began not earlier than 60 sec after the signal had reached a plateau after the previous injection. For dose-response studies, 7 mice were used.

To assess the effects of antiplatelet drugs on LDF responses, cangrelor was injected to the tail vein at doses of 0.2 and 1 mg/kg b.w. and epifibatide at doses of 0.5 and 4 mg/kg b.w. immediately before the measurement. For each dose, 7–9 mice were tested.

The curves of LDF response obtained for each injection were analyzed with the use of modified PAMA software, dedicated for the analysis of platelet aggregation curves [8].

### Experimental thrombocytopenia

To induce thrombocytopenia, mice were injected intraperitoneally with 5 μg of MwReg30 (BD Biosciences) antibodies for four consecutive days [9]. In 3 mice, blood platelet count was assayed with Scil ABC VET veterinary hematological analyzer (Horiba ABX Diagnostics, Japan). Five animals were used in LDF studies. In this experimental part, 8 mice were used.

### *In vivo* microscopy

Mice were anesthetized as described above. Three mice were injected with platelet-specific fluorescent DyLight488-labeled anti-GPIbβ antibodies (X488, Emfret, Germany) into the tail vein at a dose of 0.1 μg/g b.w., while three others were injected with rhodamine-6G at a dose of 10 μg/g b.w. The anesthetized animal was placed on the stage of an AxioExaminer epifluorescence microscope (Zeiss, Germany). The abdominal cavity was opened, and then the mesenteric bed was gently exposed with the use of cotton swabs and fixed in a chamber, before being superfused with a warmed Krebs-Henseleit buffer. ADP was administered into the abdominal vena cava, as described in the LDF measurement procedure. The observation was carried out through either 20x or 40x saline-immersion lenses. The observations were conducted in 3 different fields of a second-order mesenteric vein and artery. For the *in vivo* microscopic study, 6 mice were used.

### *Ex vivo* imaging

*Ex vivo* imaging of the fluorescence of platelets stained with the DyLight 649-labeled anti-GPIbβ antibodies (X649, Emfret, Germany) was performed using an In-vivo MS FX PRO system (Carestream Health INC., USA). Antibodies were injected into the tail vein at a dose of 0.1 μg/g b.w. After 20 minutes, ADP was injected into tail vein at a dose of 10 mg/kg b.w. Immediately after administration of ADP mice were euthanized by cervical dislocation and the lungs, livers, kidneys and spleen were isolated. The fluorescence of the probe localized in the studied organs was measured in comparison to the fluorescence of the probe accumulated in the internal organs of the mouse administered with antibody alone. The images were captured using following settings: exposure time = 30 s, f-stop = 2.8, FOV = 200, excitation filer = 650 nm, emission filter = 700 nm, no binning was applied.

The fluorescence of the probe which had accumulated within the selected tissues was measured using Carestream MI SE software (Carestream Health INC., USA). The correction for autofluorescence was performed by subtracting the total fluorescence of the tissue isolated from the mouse receiving antibody alone from that of the corresponding tissue derived from the animals treated with antibody followed by ADP. For the *ex vivo* imaging, a total of 7 mice were used (5 animals to visualize the effects of ADP and 2 to obtain a background fluorescence of antibodies).

#### Ethics statement

The studies were approved by the Local Ethical Committee on Animal Experiments, Medical University of Lodz (approval number 65/ŁB572/2011).

### Statistics

The normality of the data distributions was verified with the Shapiro-Wilk test, and variance homogeneity was tested with Levene’s and Brown-Forsyth’s test. Parametric inference tests were used for normally distributed, homoscedastic data. In some cases, transformed was used instead of raw data in order to ensure normal distributions and data homogeneity, and to use parametric tests. The significance of differences for the effect of ADP dose on LDF parameters was tested using ANOVA with repeated measures, followed by the paired Student’s t-test with Bonferroni’s correction for *post-hoc* multiple comparisons analysis. The unpaired Student’s *t*-test was employed to determine the significance of differences between two independent groups (i.e. the effect of MwReg30 antibodies on platelet count, AUC and A_max_). The ADP response curves in cangrelor- and eptifibatide-treated vs. non-treated mice were compared with a non-linear regression and analysis tool (GraphPad Prism ver. 5.0).

## Results

### LDF measurements

**Fig 2:**
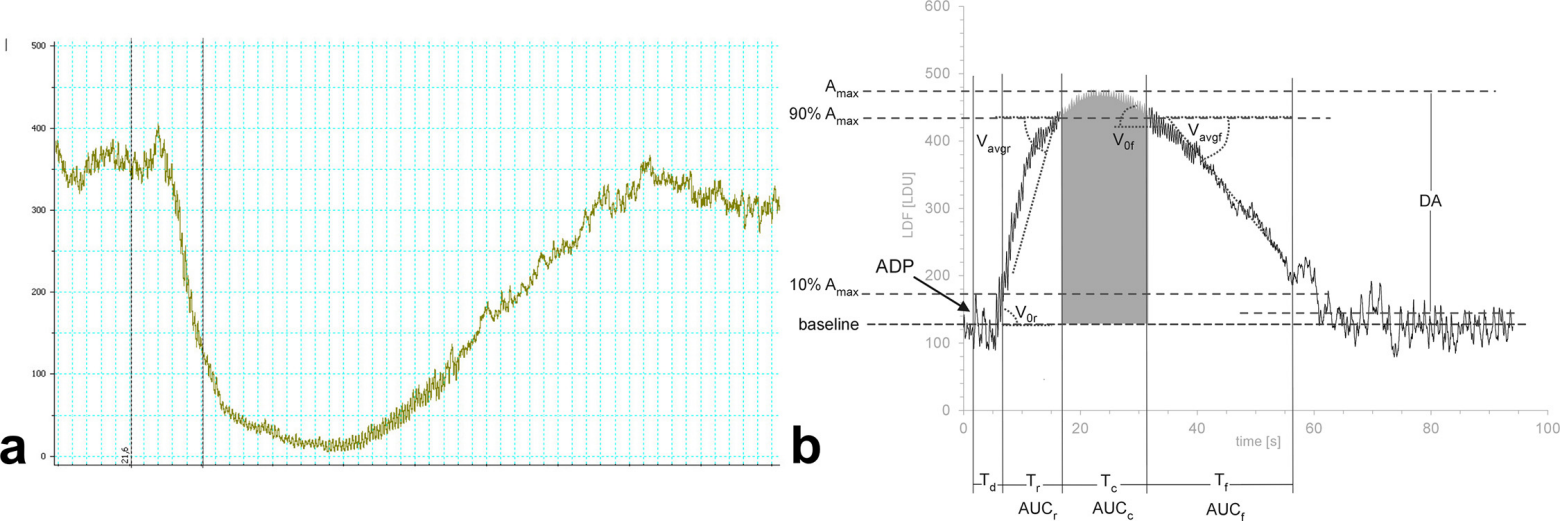
The representative trace of the LDF signal after the intravital injection of ADP to a mouse (a), parameters of the curve calculated by PAMA software, the LDU values are multiplied by -1 and an offset is added to obtain positive values (b). The following parameters characterizing the curve were calculated using PAMA software, as described in ‘*Materials and Methods*’ and are expressed in arbitrary Laser Doppler Flow units (LDU): **A**_**max**_**—**the maximum decrease in blood flow with respect to the baseline; **DA–**the maximal restoration of flow with respect to the baseline; **T**_**d**_**—**time from the injection of ADP to 10% of the maximum decrease in blood flow (A_max_); **T**_**r**_**−**time from 10% to 90% of the maximum decrease in blood flow (A_max_); **T**_**c**_**−**time from 90% of A_max_ to 90% of DA; **T**_**f**_**−**time from 90% of DA to 10% of DA; **V**_**0r**_ **–**the maximal rate of the decrease in blood flow; **V**_**Avgr**_**−**the rate of decrease averaged through T_r_; **V**_**0f**_ **–**the maximal rate of the restoration of blood flow; **V**_**Avgf**_**−**the rate of restoration averaged through T_f_; **AUC–**the area under the curve from 10% of A_max_ to 10% DA; **AUC**_**r**_**−**the area under the curve from 10% to 90% of A_max_; **AUC**_**c**_**−**the area under the curve from 90% of A_max_ to 90% of DA; **AUC**_**f**_**−**the area over the curve from 90% to 10% of DA.

The representative trace of LDF response after ADP injection is shown in **Fig 2**. Although the LDF signal is proportional to the blood flow rate, the technique does not measure absolute blood flow, expressed in velocity units. Instead, the flow rate is presented in arbitrary laser Doppler units (LDU). The figure demonstrates that injection of ADP to the abdominal vein resulted in a transient decrease of blood flow in the mesenteric vessels.

**Fig 3:**
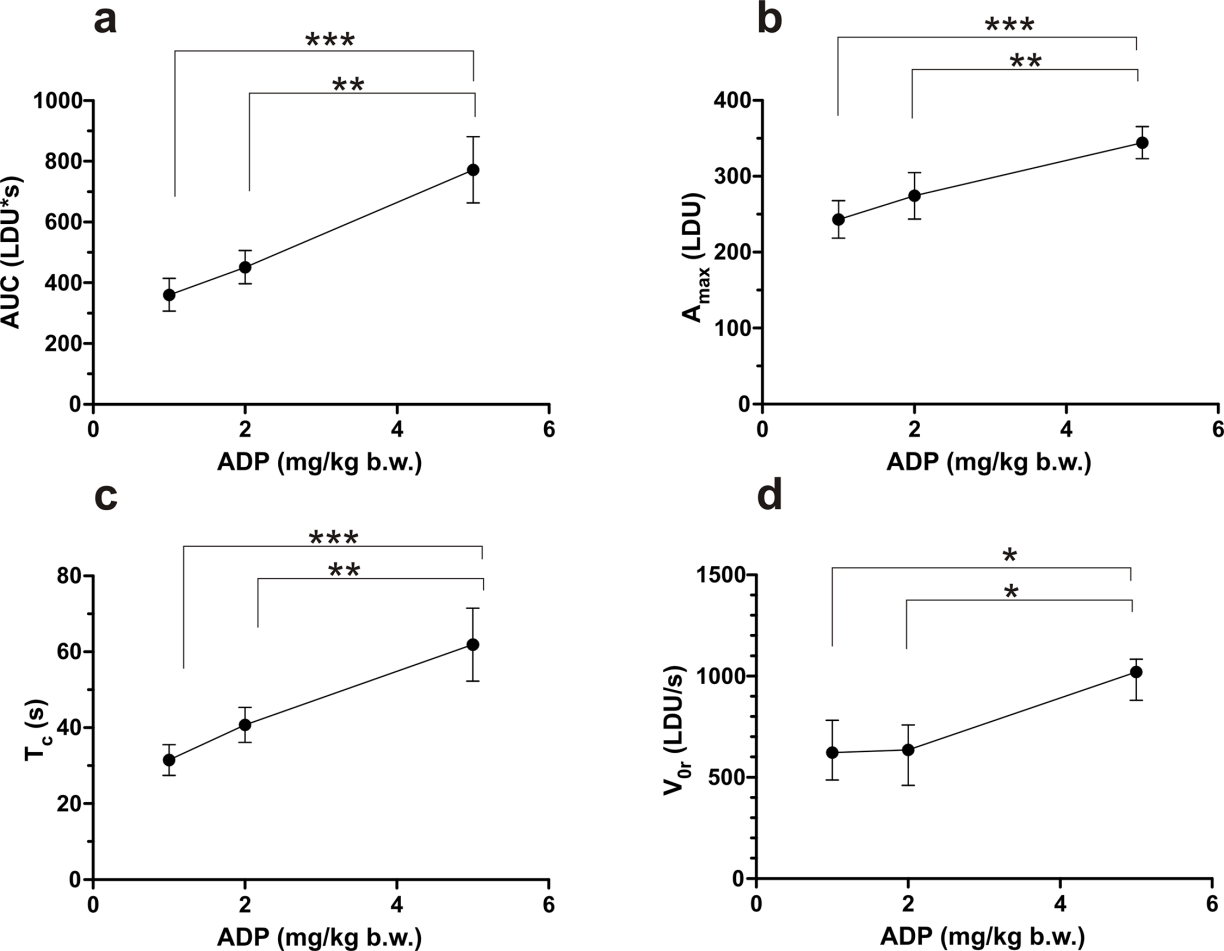
Changes in the four most discriminating parameters characterizing the LDF responses following the injections of increasing concentrations of ADP. Each dose of ADP was injected in two consecutive boluses to the same mouse (with intervals). The values represent means ± standard deviations for normally-distributed data (AUC, A_max_, T_c_) and medians with interquartile ranges for the data which significantly deviated from normal distribution (V_0r_); (n = 7). Differences on raw or transformed data (V_0_ –square, V_avgf_—square root) were tested with repeated measures ANOVA followed by a paired Student’s t test with Bonferroni’s correction for multiple comparisons. * 0.01 < p < 0.05; ** 0.001 < p < 0.01; *** p<0.001. **A**_**max**_**—**the maximum decrease in blood flow; **AUC–**the area under the curve from 10% of maximum decrease (A_max_) to 10% of the maximal restoration of flow (DA); **T**_**c**_**−**time from 90% of maximum decrease (A_max_) to 90% of value of maximal restoration of flow (DA); **V**_**0f**_ **–**the maximal rate of the restoration of blood flow.

The set of parameters characterizing each curve representing the response to a single injection of ADP was analyzed with dedicated software. These parameters, calculated for each dose of ADP, are shown in **Table 1**. To determine which ones discriminate best between the doses of the employed ADP concentrations, the calculations were analyzed by ANOVA with repeated measures. F-statistics calculated for each parameter (**Table 1**) indicated that A_max_, AUC, V_0r_ and T_c_ discriminated the used doses of ADP to the highest extent. A subsequent *post-hoc* multiple comparisons analysis revealed that the values obtained for the highest concentration of 5 mg/kg ADP were significantly higher than those recorded for the two lower doses of 1 mg/kg and 2 mg/kg; these two lower doses however, did not differ significantly from each other with respect to any of the parameters (**Fig 3).**

**Table 1:** The values of flow curve parameters characterizing the LDF response to increasing doses of ADP, calculated with the use of PAMA software. Each dose of ADP was injected subsequently two times to the same mouse. The values represent means ± standard deviations for normally-distributed data, and medians with interquartile ranges for data that was not normally distributed; (n = 7).

Parameter	ADP dose (mg/kg b.w.)			F-statistics
	1	2	5	
A_max_ (mean±SEM)	243±25	274±30	344±21	20.48
DA (mean±SEM)	233±34	264±40	331±33	5.450
T_d_ (median, IQR)	33.6 (26.2; 42.2)	24.5 (18.7; 30.5)	20.4 (17.4; 27.9)	2.924
T_r_ (median, IQR)	40.0 (24.6; 42.9)	38.1 (37.0; 43.5)	36.4 (30.4; 44.4)	5.315
T_c_ (mean±SEM)	31.5±4.0	40.8±4.6	61.9±9.6	14.96
T_f_ (mean±SEM)	107±11	118±13	161±19	7.555
V_0r_ (median, IQR)	622 (487; 781)	636 (460; 759)	1021 (881; 1083)	15.23
V_Avgr_ (mean±SEM)	364±54	356±60	468±54	5.451
V_0f_ (mean±SEM)	375±30	451±68	528±51	4.341
V_Avgf_ (median, IQR)	105 (91; 117)	110 (94; 134)	96.0 (94; 100)	2.088
AUC (mean±SEM)	361±54	451±55	772±109	17.83
AUC_r_ (mean±SEM)	78±10	98±7	130±11	8.468
AUC_c_ (mean±SEM)	129±21	185±28	357±74	12.72
AUC_f_ (mean±SEM)	154±26	168±24	285±34	12.90

Data given as mean ± SEM or median and interquartile range (lower quartile, 25% to upper quartile, 75%, IQR), depending on conformity with a normal distribution; n = 6

**A**_**max**_**—**the maximum decrease in blood flow with respect to the baseline; **DA–**the maximal restoration of flow with respect to the baseline; **T**_**d**_**—**time from the injection of ADP to 10% of the maximum decrease in blood flow (A_max_); **T**_**r**_**−**time from 10% to 90% of the maximum decrease in blood flow (A_max_); **T**_**c**_**−**time from 90% of A_max_ to 90% of DA; **T**_**f**_**−**time from 90% of DA to 10% of DA; **V**_**0r**_ **–**the maximal rate of the decrease in blood flow; **V**_**Avgr**_**−**the rate of decrease averaged through T_r_; **V**_**0f**_ **–**the maximal rate of the restoration of blood flow; **V**_**Avgf**_**−**the rate of restoration averaged through T_f_; **AUC–**the area under the curve from 10% of A_max_ to 10% DA; **AUC**_**r**_**−**the area under the curve from 10% to 90% of A_max_; **AUC**_**c**_**−**the area under the curve from 90% of A_max_ to 90% of DA; **AUC**_**f**_**−**the area over the curve from 90% to 10% of DA.

**Fig 4:**
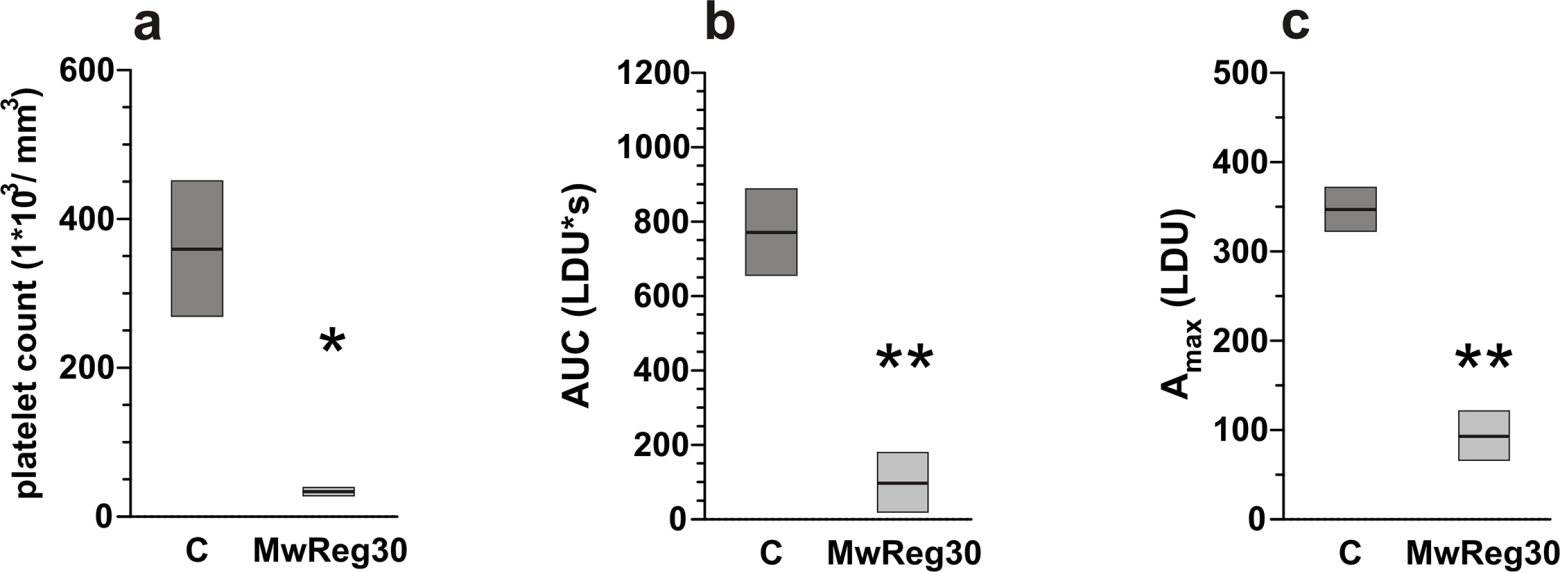
Effects of antiplatelet antibodies on blood platelet count and blood flow in circulation. (a) Platelet count in mice administered with MwReg30 antibody or saline (C). Data presented as mean ± SEM; n = 5 for control and n = 3 for MwReg30. Significance of differences tested with the unpaired Student’s t-test. (b, c) Changes in blood flow in the circulation of mice administered with MwReg30 antibody or saline (C) and subsequently given a bolus of 5 mg/kg ADP, determined from the LDF recordings and presented as AUC or A_max_. Significance of differences tested with an unpaired Student’s t-test.

To confirm that the observed responses were caused by the ADP-induced occlusions of blood vessels created by the formation of platelet aggregates in the circulating blood, three tests with the use of LDF were performed. The first addressed the model of platelet depletion. The mice that were pretreated with the MwReg30 antibodies for four consecutive days demonstrated significantly lower platelet counts (**Fig 4A**). The animals were also characterized by excessive bleeding during the preparation of mesentery and venous cannulation. To avoid the risk of animal death before completion for the platelet-depleted mice, the protocol of LDF measurements was limited to the highest dose of ADP (5 mg/kg b.w.). As shown in **Fig 4B and 4C,** the blood flow decrease in response to ADP was dramatically and significantly lower than that observed in mice not treated with the MwReg30 antibody.

**Fig 5:**
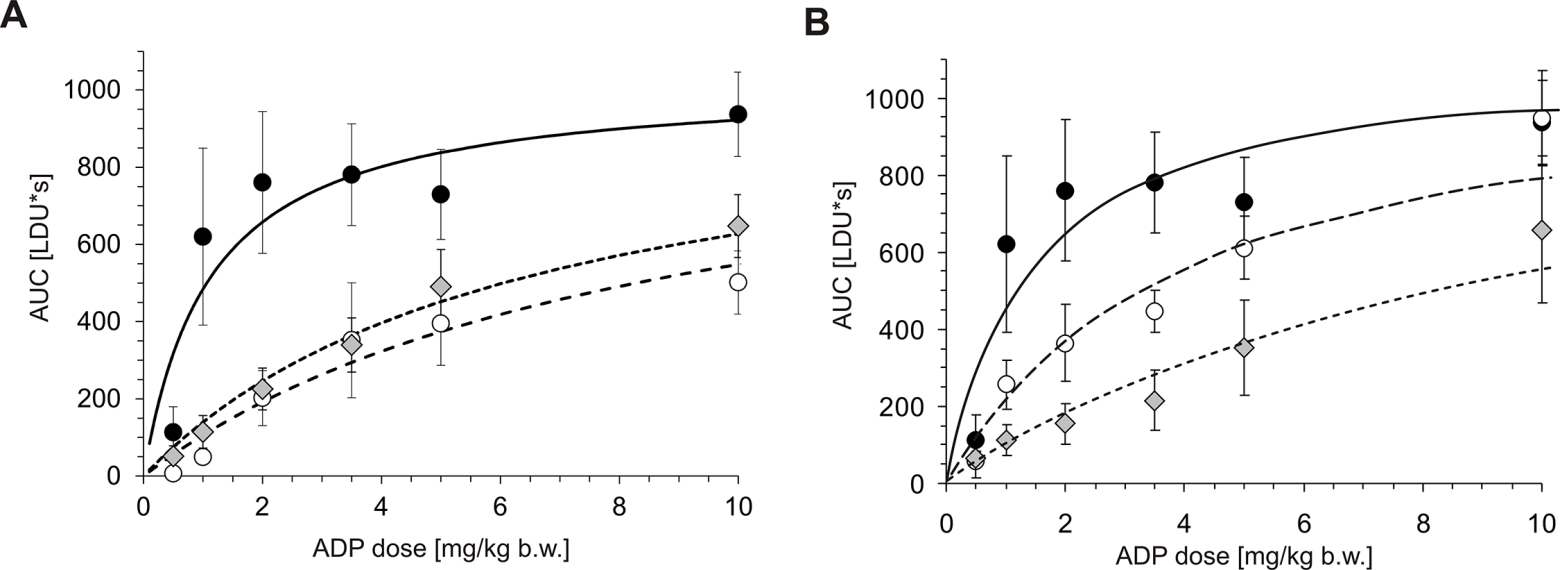
Effects of cangrelor and eptifibatide on the dose-dependent changes of AUC and A_max_ of the LDF recordings in mice administered with the increasing doses of ADP. Data is presented as mean ± SEM; n = 7–9 mice. Animals were pre-injected with various doses of either cangrelor (A) (black circle- 0 mg/kg b.w., open circle- 0.2 mg/kg b.w., grey diamond- 1 mg/kg b.w.) or eptifibatide (B) (black circle- 0 mg/kg b.w., open circle- 0.5 mg/kg b.w., grey diamond- 4 mg/kg b.w.), and then administered with boluses of increasing doses of ADP. The curve was fitted to the one-site binding non-linear regression curve (GraphPad Prism ver. 5.0): $y=\frac{a\;*\;x}{b+x}$, where ‘a’ is the maximum achievable AUC and ‘b’ is the concentration of the agonist (ADP), at which the response reaches 50%. Cangrelor: shared a = 1025 ± 111, b _control_ = 1.12 ± 0.43; b _cangrelor 0.2 mg/kg b.w._ = 8.69 ± 2.37; b _cangrelor 1 mg/kg b.w._ = 6.34 ± 1.83; *p* < 0.0001 for comparing of all curves, *p* < 0.001 for b_control_ < b_cangr 0.2_ = b_cangr 1_; based on extra sum-of-squares *F*-statistics and the multiple comparison testing with the use of Bonferroni’s correction. Eptifibatide: shared a = 1104 ± 129, b _control_ = 1.38 ± 0.54; b _eptifibatide 0.5 mg/kg b.w._ = 3.87 ± 1.23; b _eptifibatide 4 mg/kg b.w._ = 9.66 ± 2.94; *p* < 0.0001 for comparing of all curves, *p* < 0.001 for b_control_ < b_cangr 0.5_ < b_cangr 4_; based on extra sum-of-squares *F*-statistics and the multiple comparison testing with the use of the Bonferroni’s correction.

The second experiment examined the responses to ADP in mice pre-injected with cangrelor, the antagonist of P_2_Y_12_ receptor, while the third examined the response in mice pre-injected with eptifibatide, an inhibitor of platelet aggregation. Both inhibitors significantly suppressed blood platelet response to the agonist in mice administered with increasing doses of ADP: by 87.1 ± 27.3% and 82.4 ± 28.8%, respectively in the presence of either 0.2 or 1 mg/kg cangrelor, and by 64.3± 31.8% and 85.7 ± 30.5%, respectively in the presence of either 0.5 or 4 mg/kg eptifibatide (**Fig 5).** Thus, the results of the platelet depletion and platelet function inhibition models confirm that the decrease in blood flow occurring in response to ADP, as measured by LDF, is platelet-dependent.

### *In vivo* and *ex vivo* visualization of the platelet aggregates

**Fig 6:**
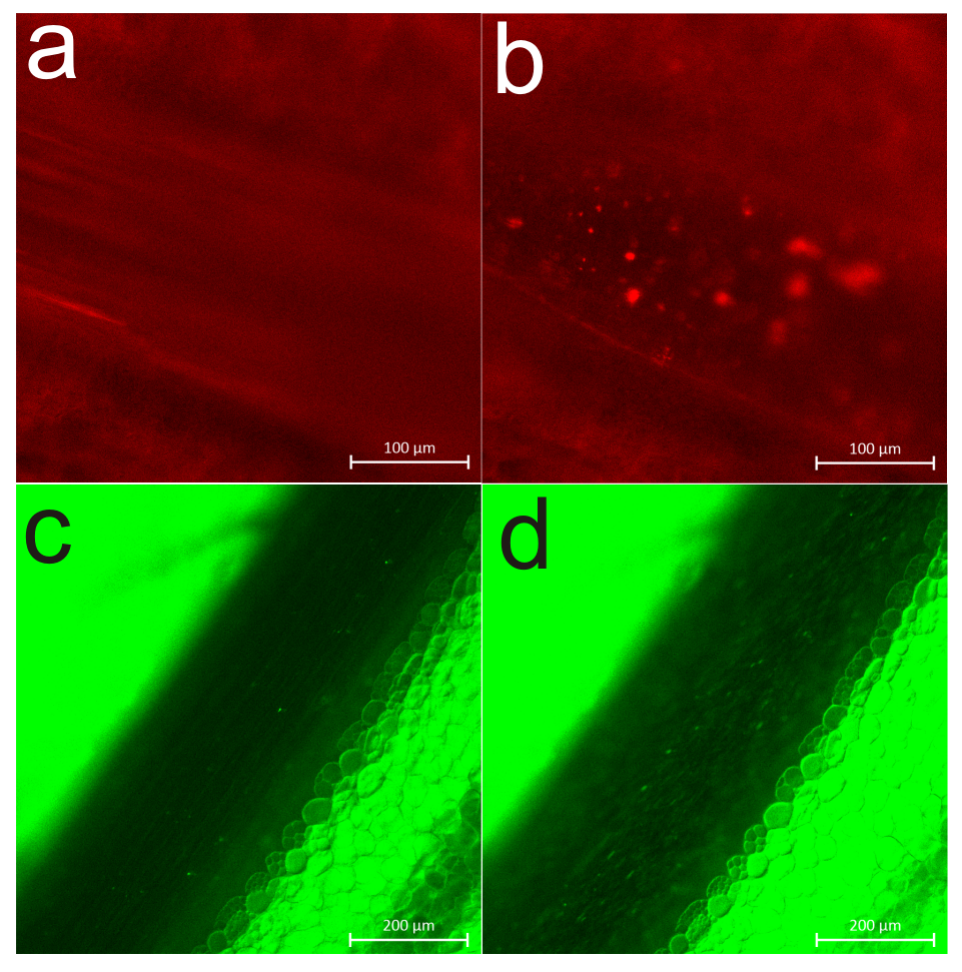
Representative picture of a mesenteric vessel prior to (a, c) and after the reduction of blood flow upon ADP injection (10 mg/kg b.w.) (b, d). Animals were pre-injected with rhodamine (a, b) or DyLight488-labeled anti-GPIbβ antibodies (c, d).

Intravital microscopy revealed that injections of ADP to the vena cava caused a transient decrease in blood flow in the mesenteric vessels, which remained consistent with LDF responses. The fluorescent objects flowing in the blood stream were seen as traces or smears (**Fig 6A and 6C)** and when the flow decreased or stopped, they were seen as separate objects (**Fig 6B and 6D).** However, during the flow cessation period, no aggregates was found to accumulate in the examined mesenteric vessels, as visualized with either rhodamine 6G staining **(Fig 6B)** or platelet-specific antibodies **(Fig 6D)**.

**Fig 7:**
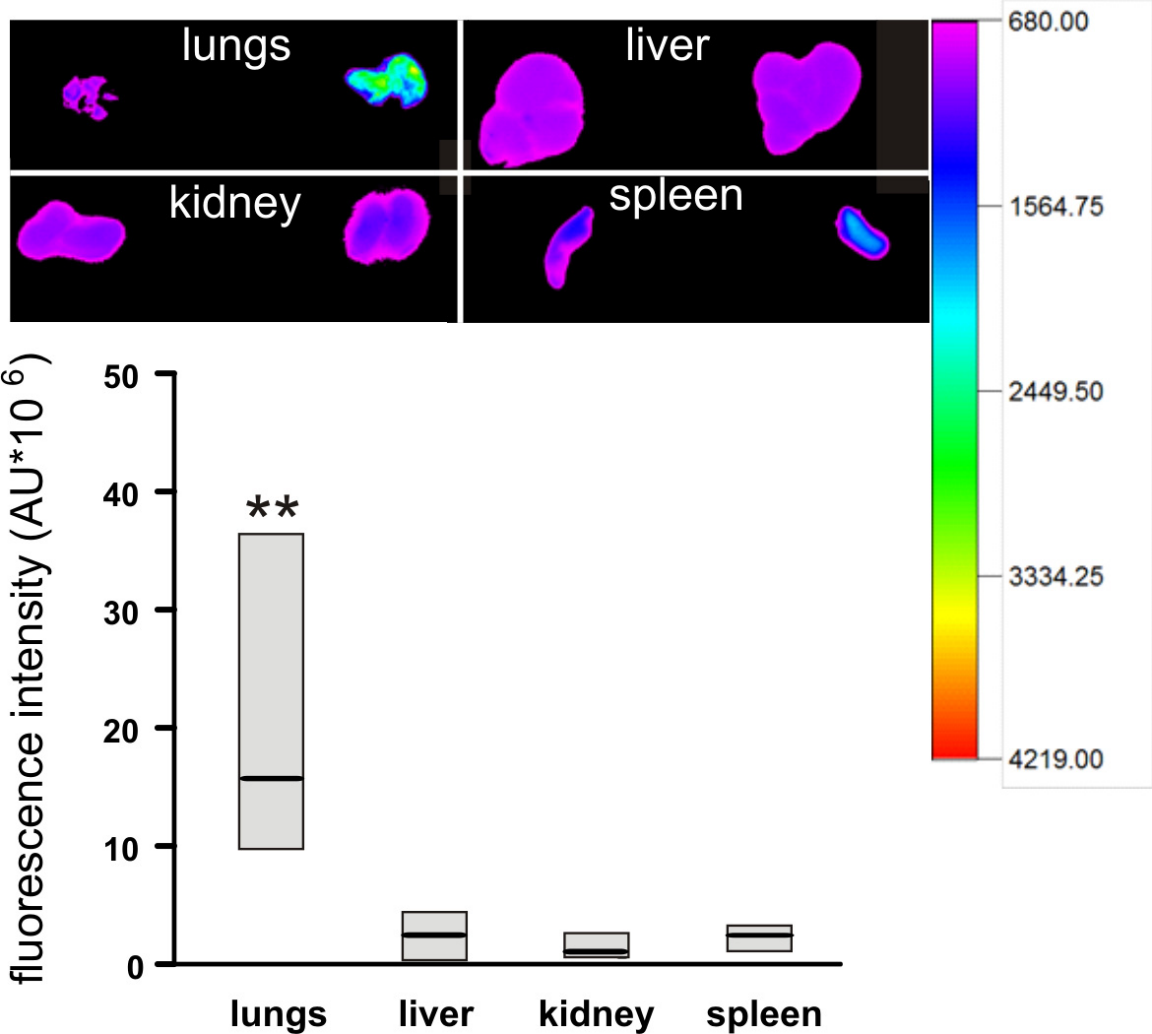
Accumulation of platelet aggregates labelled with DyLight 649 anti-GPIbβ antibodies in selected organs of mice injected with ADP (10 mg/kg b.w.). The picture shows the representative organs excised from the mouse injected with antibodies (left) and antibodies followed by ADP (right). For a quantitative analysis, the total fluorescence of organs originating from animals not injected with ADP was subtracted from the fluorescence values recorded for ADP-injected animals. Such ‘corrected’ values are presented as median with IQR (n = 5). The paired t-test with Bonferroni’s correction for multiple comparisons was used to determine the significance of differences between control and ADP-administered mice for lungs and other organs.

The above results suggest that the formation of aggregates takes place in other organs. To verify the site where the platelet accumulation predominantly occurs, the examination moved to the lungs, livers, kidneys and spleens of the animals pre-injected with DyLight649 anti-GPIbβ fluorescent antiplatelet antibodies and consecutively treated with 10 mg/kg b.w. ADP. The strongest fluorescent signal of the antibody after intravenous administration of ADP was localized in the lungs, while the fluorescence in the liver, kidney and spleen was similar to the fluorescence measured for the corresponding tissues isolated from mice receiving antibody alone **(Fig 7)**. A comparison between the organs confirmed that the greatest fluorescence was found in the lungs.

## Discussion

The presented paper describes for the first time a new approach for the quantification of pulmonary thromboembolism in mice. Its primary intention was to establish a method in which blood platelet reactivity could be assayed *in vivo* by the injection of an agonist causing reversible platelet aggregation in the peripheral circulation. This aggregation was to be monitored by measuring the changes in blood flow with the use of laser Doppler flowmetry. The chosen agonist was ADP, which is known to induce reversible aggregation.

Intravenous injection of ADP was found to result in the dose-dependent, reversible cessation of the blood flow, which could be detected in the mesenteric vessels with the use of laser Doppler flowmetry. As shown in platelet-depleted, cangrelor-treated and eptifibatide-treated animals, the flow cessation was attributable to the platelet aggregation. However, contrary to our expectations, *in vivo* microscopic observations revealed that reduced blood flow in the branches of mesentery vessels was not accompanied by the build-up of aggregates in these vessels. In turn, platelet accumulation was found to be greatest in the lung. These results are in agreement with previously published observations [4]. Such an effect can be explained by the fact that the lungs are the first capillary system in the route of venous blood exposed to ADP and as this agonist is of a relatively short-acting nature, the entire process of aggregation and disaggregation takes place predominantly in the lungs.

This conclusion also explains some discrepancies between responses to ADP observed in the present study and those measured elsewhere in animals with radiolabeled platelets. Maximum accumulation of radiolabeled platelets was reported to range from 15 to 30 s after ADP injection [4,7], while the decrease in blood flow was seen to reach its maximum after a median time of 40 s (**Table 1)**. It is not surprising that platelet aggregate formation is observed before it reaches a level which is sufficient to result in decreased blood flow.

The fact that changes in blood flow measured in distant parts of the body are secondary to platelet aggregation in lungs is also demonstrated by the doses of ADP needed to generate a measurable effect. The doses of ADP sufficient to induce a measurable accumulation of radiolabeled platelets in lungs were reported to be as low as 0.4 μg/kg b.w. [4] and the doses effectively used in the experiments ranged between 15 and 400 μg/ kg b.w. [4,7]. In our approach, the lowest dose to result in a measurable flow reduction was 500 μg/kg b.w. Apparently, the formation of aggregates induced by lower doses of ADP is not sufficient to result in the depression of the blood flow measured in the mesentery.

Taking the above into account, one could suggest a more straightforward approach to creating a similar effect, i.e. to inject the agonist to a vessel which supplies blood to the same vascular bed at which the LDF probe is located. This approach is however, impossible in the case of the lungs. The main limitation of our experimental setup, is that it is extremely sensitive to any movements of organs or tissues. The movements of the tissue generate LDF signal which is indistinguishable from that resulting from flowing blood and thus complicates analysis, or even masks minor responses. Thus, the respiratory movements of lungs make them rather unsuitable subjects for LDF measurements. For this reason, the measurement of the LDF response for intravenous ADP injection has to take place in a vascular bed distant from the one where aggregation actually occurs. Therefore, it is important to choose the correct vascular bed for the experiment. The mesentery was chosen for the present study for two reasons: it is relatively easily accessible and it can be firmly fixed, which limits tissue movement and thus allows a signal devoid of noise to be acquired. Although non-invasive measurement was also tested in the paw and the ear, responses were observed only for the highest doses of ADP.

This obvious limitation of the method, i.e. the measurement of effects secondary to occlusion occurring in the lung, can be circumvented by two possible routes. One may be the intravenous injection of an agonist, which would induce slower and more gradual activation of circulating platelets than in the case of ADP, and thus, would allow the formation of aggregates after the passage of circulating platelets through the pulmonary circulation. The other might be to inject the agonist into an artery supplying a particular vascular bed. This approach would be more challenging due to the relatively smaller dimensions of arteries. In the case of mice, the femoral artery could be considered as the site of agonist injection and a lower limb as the site of measurements. These optional solutions certainly deserve further testing in the future.

Despite these limitations, our method has two immense advantages over that which utilizes radiolabeled platelets, in that it does not require radioisotopes and it does not demand the extravasation of platelets. The latter is particularly important for two reasons. One is that the researcher avoids the risk of artefactual activation of platelets, and the other is that a donor animal is not required: the need for such animals may become a considerable limitation when expensive genetic models are to be employed.

As shown by the decreased responses demonstrated in the animals pretreated with cangrelor or eptifibatide, the method is suitable for the *in vivo* screening of the activities of antiplatelet drugs. It can be specifically useful in the case of testing of pro-drugs which, unlike cangrelor, have to be metabolized prior to becoming biologically active.

## Conclusions

Intravenous injection of ADP in mice results in the dose-dependent decrease of blood flow attributable to blood platelets, which can be measured in mesenteric vessels by means of laser Doppler flowmetry. Our *in vivo* and *ex vivo* observations exclude the possibility that the decrease is caused by direct occlusions of mesenteric vessels and that it is secondary to occlusions occurring in the lung. We have also proven that the method discriminated cangrelor-treated and eptifibatide-treated mice from vehicle-treated animals, and on this basis, we suggest that it can be useful for *in vivo* screening for antiplatelet drug candidates.

## Supporting Information

S1 Fig Representative curve of LDF response to ADP in animal with thrombocytopenia induced by MwReg30 antibodies (a), curve as analyzed by PAMA software (b). (TIF)

S2 Fig Representative curve of LDF responses to ADP in animal pretreated with 1 mg/kg cangrelor (a), curve as analyzed by PAMA software (b). (TIF)

S3 Fig Representative curve of LDF responses to ADP in animal pretreated with 4 mg/kg eptifibatide (a), curve as analyzed by PAMA software (b). (TIF)
