# Correction: Quantification of the Blood Platelet Reactivity in the ADP-Induced Model of Non-Lethal Pulmonary Thromboembolism in Mice with the Use of Laser Doppler Flowmetry

**DOI:** 10.1371/journal.pone.0149829

**Published:** 2016-02-17

**Authors:** The PLOS ONE Staff

## Notice of Republication

This article was republished on February 1, 2016, to correct errors in figure captions that were introduced during the typesetting process. The publisher apologizes for the errors. Please download this article again to view the correct version. The originally published, uncorrected article and the republished, corrected articles are provided here for reference.

## Supporting Information

S1 File Originally published, uncorrected article. (PDF)

S2 File Republished, corrected article. (PDF)
