# Supplementary figures and images for: Quantification of the Blood Platelet Reactivity in the ADP-Induced Model of Non-Lethal Pulmonary Thromboembolism in Mice with the Use of Laser Doppler Flowmetry
Source: PLoS One. 2016 Jan 11;11(1):e0146346. doi: 10.1371/journal.pone.0146346 (PMC4713441; doi:10.1371/journal.pone.0146346)

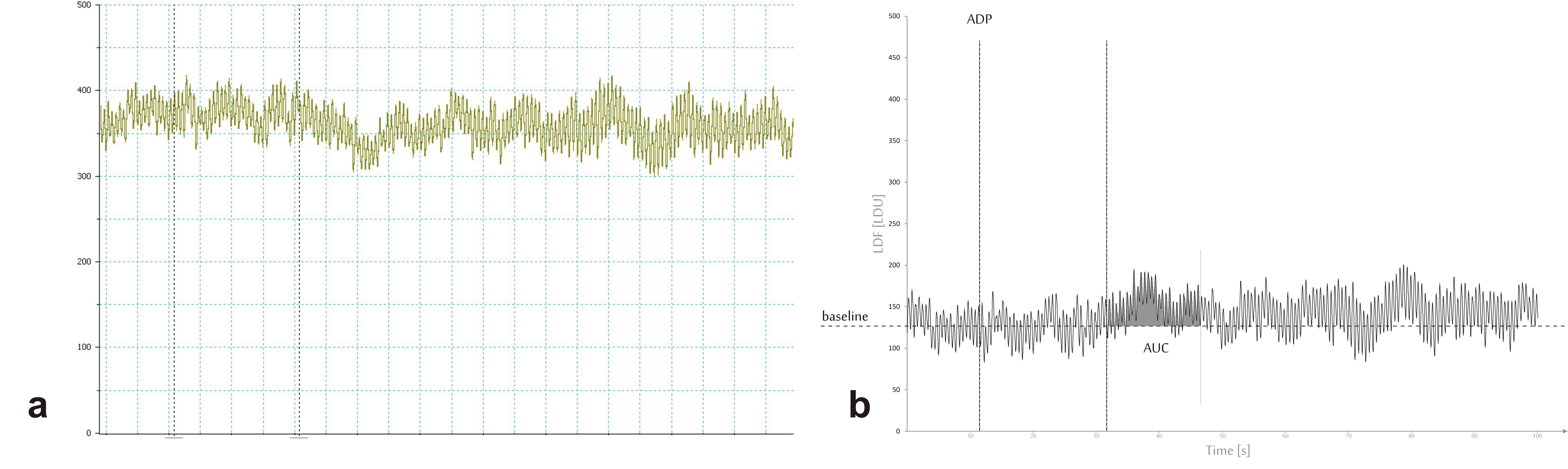

Supplement: S1 Fig — (TIF) [file pone.0146346.s001.tif]

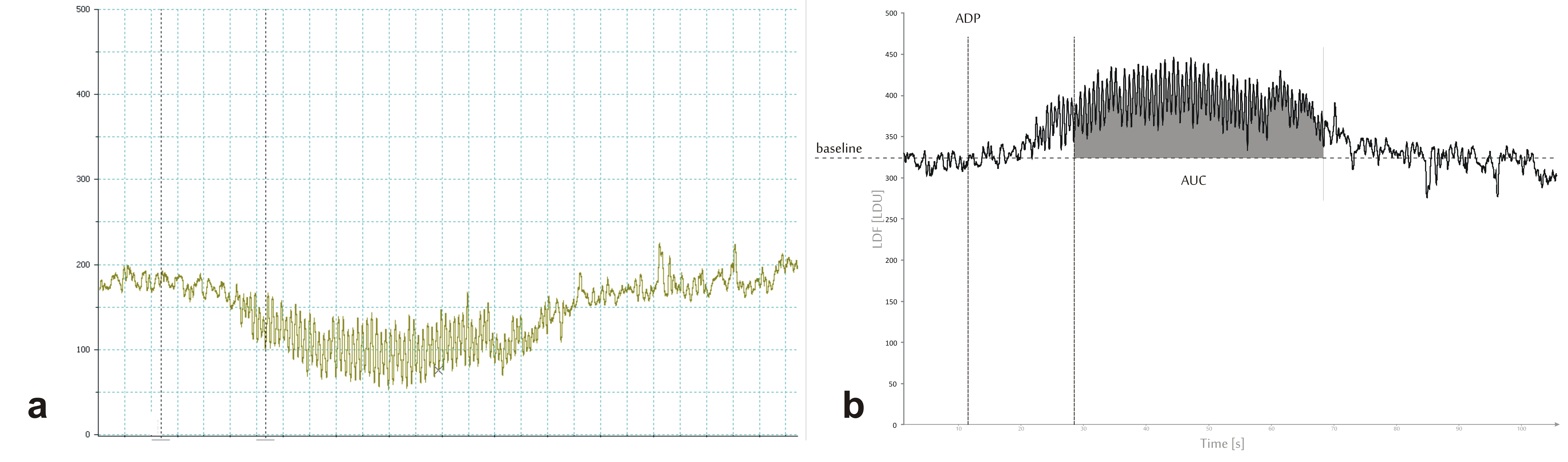

Supplement: S2 Fig — (TIF) [file pone.0146346.s002.tif]

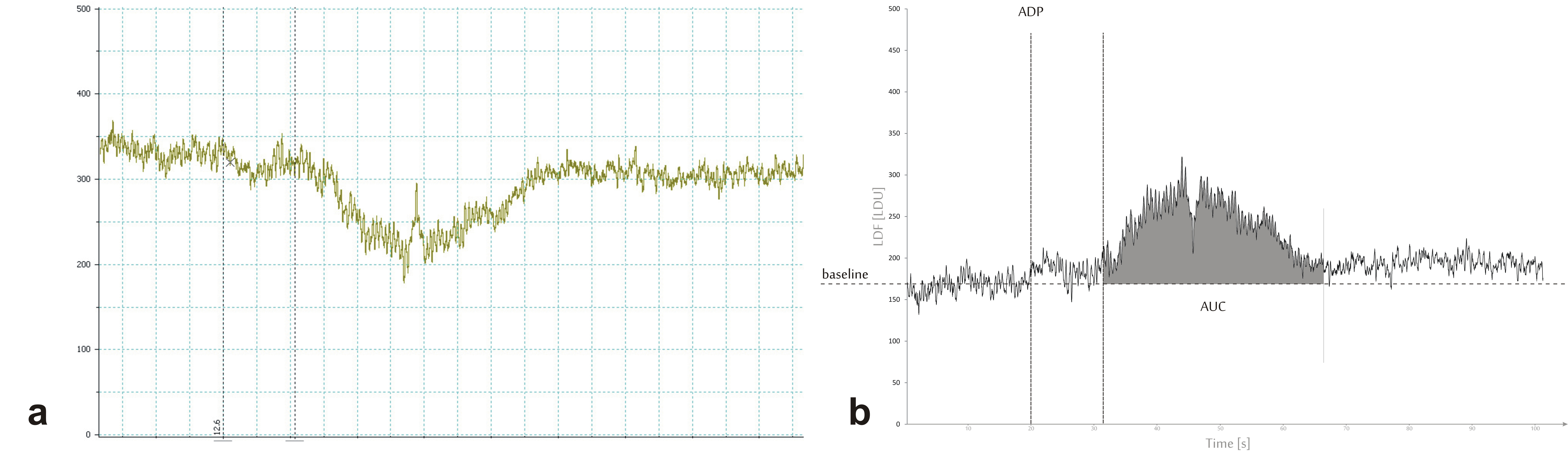

Supplement: S3 Fig — (TIF) [file pone.0146346.s003.tif]
